# Incidence of breakthrough COVID-19 in patients with hematological disorders who received pre-exposure prophylaxis with tixagevimab-cilgavimab: a retrospective study in Japan

**DOI:** 10.1038/s41409-023-02019-y

**Published:** 2023-06-17

**Authors:** Mizuki Haraguchi, Hisashi Yamamoto, Otoya Watanabe, Takashi Sakoh, Keiko Ishida, Sho Ogura, Masayo Katoh-Morishima, Yuki Taya, Aya Nishida, Daisuke Kaji, Shinsuke Takagi, Go Yamamoto, Naoyuki Uchida, Hideki Araoka

**Affiliations:** 1 Department of Infectious Diseases, Toranomon Hospital, Tokyo, Japan; 2 Department of Hematology, Toranomon Hospital, Tokyo, Japan; 3 Okinaka Memorial Institute for Medical Research, Tokyo, Japan

**Keywords:** Epidemiology, Infectious diseases

**TO THE EDITOR:**

Since SARS-CoV-2 first emerged in China in December 2019 [1], it has rapidly spread worldwide, causing a global pandemic featuring numerous waves with changing dominant variants [2]. Immunocompromised patients are a high-risk population for severe COVID-19. Vaccination is a reasonable approach to preventing COVID-19 and reducing the risk of severe disease in immunocompetent people [3]. However, a suboptimal humoral immune response to SARS-CoV-2 vaccination has been observed in patients with hematological disorders [4]. Tixagevimab/cilgavimab (Tix/Cil) is a combined SARS-CoV-2 neutralization antibody targeting SARS-CoV-2 spike protein and has shown preventive effects against COVID-19 [5]. In Japan, Tix/Cil has been offered to specific immunosuppressed patients from August 2022. Some recent studies have reported the incidence of breakthrough COVID-19 in people administered Tix/Cil [6–8], but the COVID-19 pandemic situation varies from region to region and period to period, with differences in the major SARS-CoV-2 variant, vaccination status, and Tix/Cil dose. A 150-mg dose of each of Tix/Cil was used at first in the United States but, because the in vitro data suggested a decreased affinity of Tix/Cil for Omicron variants, the FDA recommended an increase in both doses to 300 mg. Some studies have used the IgG titer to select patients who will likely have an insufficient vaccination response [7–9], but the serum neutralization antibody for SARS-CoV-2 is not measured in daily practice in Japan. The collection of real-world data would be valuable for devising a strategy for managing the COVID-19 pandemic in immunosuppressed patients.

To evaluate the incidence of breakthrough COVID-19 in Japan, we performed a retrospective, single-center analysis of consecutive 267 patients with hematological disorders who received 300 mg each of Tix/Cil at Toranomon Hospital from October 2022 to January 2023. Tix/Cil was administered as appropriate to patients in line with guidance from the Ministry of Health, Labour, and Welfare of Japan. This guidance covered patients with primary immunodeficiency and those who had received B cell-depleting therapies within the previous year, Bruton’s tyrosine kinase inhibitors (BTKi), chimeric antigen receptor-T (CAR-T) therapy, and immunosuppressive treatment after hematopoietic stem cell transplantation (HSCT). In addition, we also included patients who underwent HSCT after the administration of Tix/Cil. We excluded patients who had not visited the hospital after the administration of Tix/Cil by the end of March 2023. Finally, 257 patients were included in this study. We reviewed their medical records to collect data on age, underlying hematological disorders, treatment details, history of COVID-19, and any episode of COVID-19 after Tix/Cil administration and the clinical course. The patients’ characteristics are shown in Table 1. The median age was 60 years (range, 21–89) and 148 were male (58%). The median follow-up period from Tix/Cil administration was 73 days (interquartile range, 49.75–91). In total, 148 patients underwent HSCT, with 61% of them having an interval between HSCT and Tix/Cil administration of less than 1 year. In addition, 60 (23.3%) and 11 (9.3%) patients received anti-CD20 antibody drugs and CAR-T therapy within the previous year, respectively. Overall, 141 patients (51%) had received 3 or more doses of the SARS-CoV-2 vaccine at the time of Tix/Cil administration. According to the medical records, 18 patients contracted COVID-19 after Tix/Cil administration (Supplementary Table) and the cumulative infection rates on days 30 and 100 were 4.3% and 8.2%, respectively (Fig. 1). COVID-19 severity was defined according to the COVID-19 treatment guidelines of the US National Institutes of Health. Among the infected patients, 1 had a positive quantitative antigen test result on screening for SARS-CoV-2 at admission and was considered asymptomatic. The severity of COVID-19 was mild in 11 patients, moderate in 2, and severe in 4. Twelve patients were admitted to the hospital (mild in 6 patients, moderate in 2, and severe in 4). Patients with mild or moderate COVID-19 were treated with nirmatrelvir/ritonavir or remdesivir and no patients progressed severe disease. Four patients required conventional oxygen therapy and all of them received immunosuppressive agents such as dexamethasone. Consequently, no patients died of COVID-19 in this study. The day of COVID-19 onset was available in 17 patients, and the median time between Tix/Cil administration and COVID-19 onset was 25 days (range, 1–93). In particular, 6 patients developed COVID-19 within 14 days. We performed univariate analysis to examine the relationship of the incidence of COVID-19 with age older than 60 years, sex, 3 or more vaccine doses, HSCT, and anti-CD20 antibody drugs, but there were no significant findings.

**Table 1:** Characteristics of the patients included in this study.

Characteristic	*N* = 257
Age, median (range), y	60 (21, 89)
Male sex	148 (58%)
Underlying disease	
Myeloid malignancy	108 (42%)
Lymphoid malignancy	124 (48%)
Others	25 (9.7%)
HSCT	148 (58%)
Source of HSCT	
AutoPBSCT	16 (11%)
CBT	95 (64%)
Related HSCT	20 (14%)
Unrelated HSCT	17 (11%)
Interval between HSCT and Tix/Cil	
Less than 1 year	90 (61%)
More than 1 year	57 (39%)
CAR-T	11 (0.43%)
Anti-CD20 drug	59 (23%)
Anti-CD22 drug	2 (0.78%)
Anti-CD19 drug	11 (4.3%)
BTKi	9 (3.5%)
Administered as outpatient	127 (49%)
Vaccine status	
Unvaccinated	35 (14%)
1 dose	7 (2.7%)
2 doses	52 (20%)
3 or more doses	141 (55%)
Unknown	22 (8.6%)
History of COVID-19	31 (12%)
Days between COVID-19 and Tix/Cil, median (range)	98 (12, 512)

Data are *n* (%), unless otherwise indicated.

*HSCT* hematological stem cell transplantation, *PBSCT* peripheral blood stem cell transplantation, *CBT* cord blood transplantation, *CAR-T* chimeric antigen receptor-T, *BTKi* Bruton’s tyrosine kinase inhibitor.

**Fig. 1:**
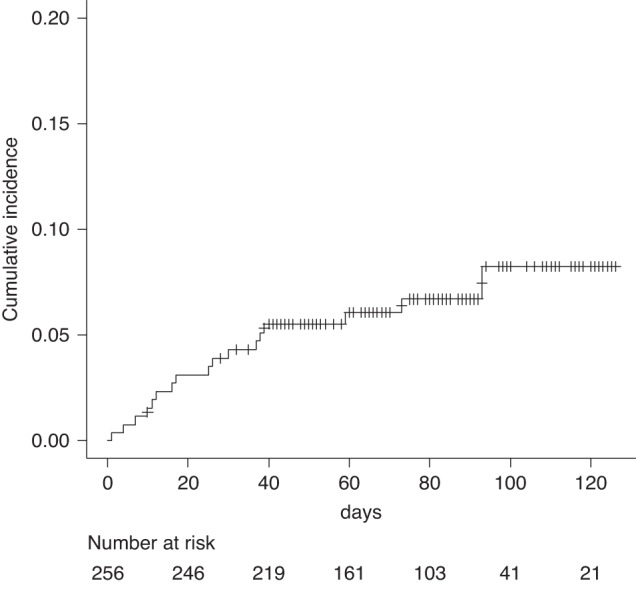
Cumulative incidence of breakthrough COVID-19. Daily cumulative incidence curve of breakthrough COVID-19 using the cumulative incidence function.

In this study, we demonstrated a 7.5% incidence (18 of 257) of breakthrough COVID-19 in our cohort of patients with hematological disorders who received pre-exposure prophylaxis with Tix/Cil. To our knowledge, this is the first real-world data evaluating the incidence of breakthrough COVID-19 in this patient population in Japan. Recent studies have reported that the incidence of breakthrough COVID-19 was between 11 and 14% in patients with hematological malignancy [7, 8]. Our rate is similar to those results, but there were differences in the Tix/Cil dose and the epidemic situation of COVID-19, such as the number of confirmed daily cases of COVID-19. The peak of the number of confirmed daily infected patients in Tokyo was between November 2022 and January 2023 [10]. This was during the study period, suggesting that our result can be considered a real-world experience in Japan. The neutralization activity of Tix/Cil depends on the SARS-CoV-2 variant [11, 12]. Although we did not perform genome sequencing of SARS-CoV-2 in this study, the most dominant strain of SARS-CoV-2 during the study period was BA.5, suggesting that the impaired activity of Tix/Cil for SARS-CoV-2 neutralization affected the incidence of breakthrough infection [13]. Because the health care system in Japan is responsive to the needs of patients with high-risk factors related to increased mortality, 12 patients (67%) were hospitalized. However, in line with the previous reports, we did not observe mortality due to COVID-19, suggesting the preserved efficacy of Tix/Cil against disease progression in Japan [8, 9].

This study has some limitations. First, this is a retrospective study without a control group. Accordingly, it is difficult to evaluate the absolute efficacy of Tix/Cil from our results. In addition, the patient characteristics associated with breakthrough infection could not be shown statistically, possibly due to the limited number of patients. Second, the patients who developed COVID-19 within 14 days from Tix/Cil administration may not truly represent a breakthrough infection because sufficient efficacy of Tix/Cil would not have been achieved. Tix/Cil is administered intramuscularly and shows a plasma concentration that is sustained for over 90 days. However, their concentrations do not immediately increase, in contrast to intravenous administration, and the peak concentration is achieved about 14 days after administration, suggesting that the patients who developed COVID-19 within 14 days after Tix/Cil administration would not have a sufficient concentration for inhibiting the occurrence of COVID-19 [14]. Although future studies are needed to declare the relation between COVID-19 incidence and the days from administration of Tix/Cil, patients may need to maintain cautious behavior to avoid infection during this period, and Tix/Cil should be administered well in advance of the peak of the epidemic. Third, we first administered Tix/Cil to inpatients, and then outpatients. The risk of COVID-19 differs between inpatient and outpatient settings. Moreover, undiagnosed or unreported cases may be present because this study is based on medical records. These factors may have contributed to an underestimation of breakthrough infections.

In conclusion, we performed a retrospective study to evaluate the incidence of COVID-19 in patients with hematological disorders after pre-exposure prophylaxis using Tix/Cil in Japan. Among the 257 patients analyzed, 18 developed COVID-19, but no patients died, suggesting the effectiveness of Tix/Cil for preventing the development of critical COVID-19. This is the first report describing the real-world experience during the Omicron era in Japan. Our results thus indicate that pre-exposure prophylaxis, vaccination, and early intervention for COVID-19 are effective strategies for decreasing the mortality risk in immunocompromised patients. We should pay attention to factors that will influence the effective management strategy for the COVID-19 pandemic, such as changes in the virulence or immune escape efficacy of dominant variants, and we need to continue modifying the therapeutic strategy based on research results.

## Supplementary information

Supplemental Table

## Data Availability

The data generated and analyzed in this article are available from the corresponding author upon reasonable request.
