# Supplementary material for: Incidence of breakthrough COVID-19 in patients with hematological disorders who received pre-exposure prophylaxis with tixagevimab-cilgavimab: a retrospective study in Japan
Source: Bone Marrow Transplant. 2023 Jun 17;58(9):1051–3. doi: 10.1038/s41409-023-02019-y (PMC10471491; doi:10.1038/s41409-023-02019-y)
Supplement: Supplementary file 1 — Supplemental Table [file 41409_2023_2019_MOESM1_ESM.pptx]

## Slide 1
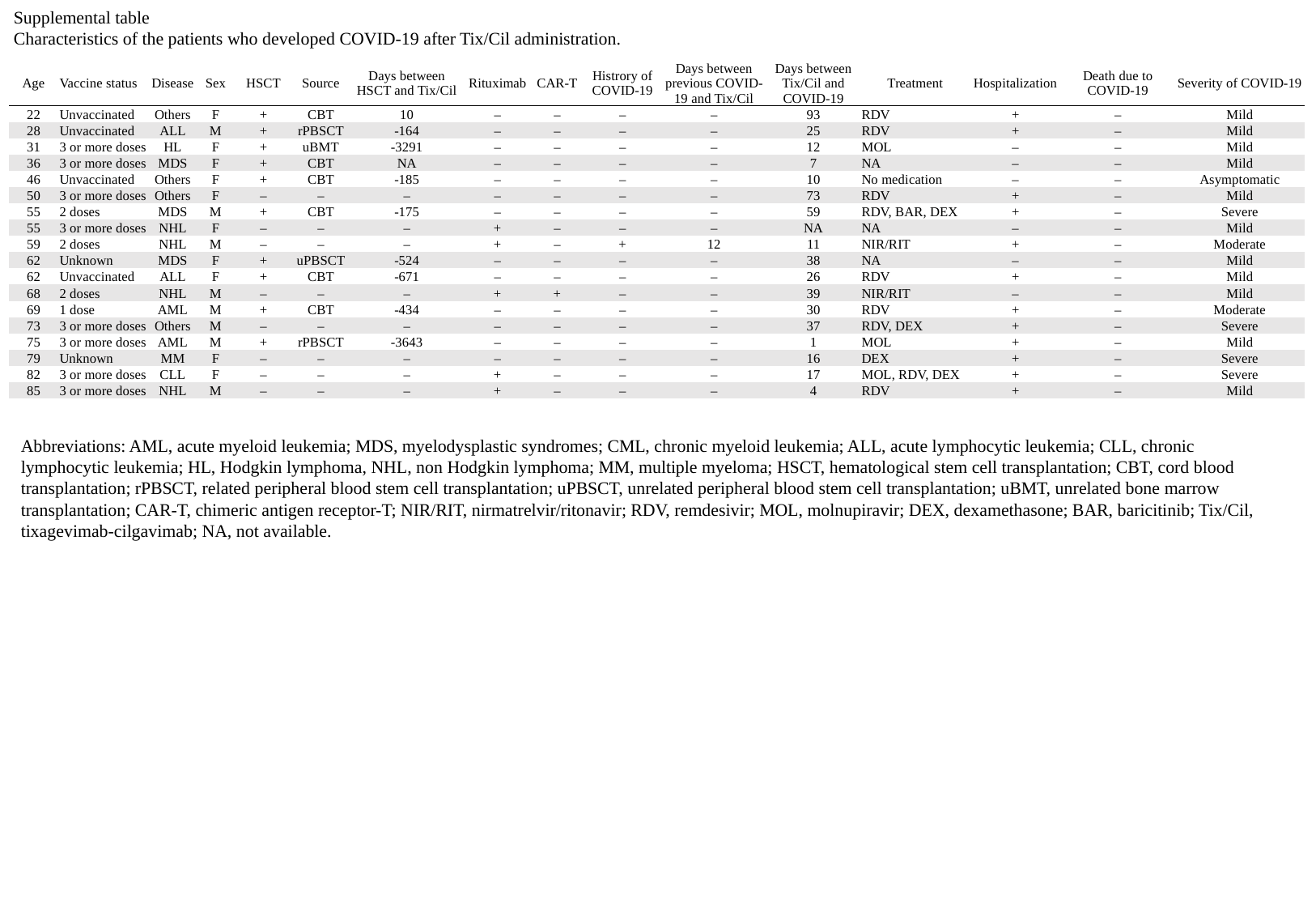

Supplemental table
Characteristics of the patients who developed COVID-19 after Tix/Cil administration.
| Age | Vaccine status | Disease | Sex | HSCT | Source | Days between HSCT and Tix/Cil | Rituximab | CAR-T | Histrory of COVID-19 | Days between previous COVID-19 and Tix/Cil | Days between Tix/Cil and COVID-19 | Treatment | Hospitalization | Death due to COVID-19 | Severity of COVID-19 |
| --- | --- | --- | --- | --- | --- | --- | --- | --- | --- | --- | --- | --- | --- | --- | --- |
| 22 | Unvaccinated | Others | F | + | CBT | 10 | – | – | – | – | 93 | RDV | + | – | Mild |
| 28 | Unvaccinated | ALL | M | + | rPBSCT | -164 | – | – | – | – | 25 | RDV | + | – | Mild |
| 31 | 3 or more doses | HL | F | + | uBMT | -3291 | – | – | – | – | 12 | MOL | – | – | Mild |
| 36 | 3 or more doses | MDS | F | + | CBT | NA | – | – | – | – | 7 | NA | – | – | Mild |
| 46 | Unvaccinated | Others | F | + | CBT | -185 | – | – | – | – | 10 | No medication | – | – | Asymptomatic |
| 50 | 3 or more doses | Others | F | – | – | – | – | – | – | – | 73 | RDV | + | – | Mild |
| 55 | 2 doses | MDS | M | + | CBT | -175 | – | – | – | – | 59 | RDV, BAR, DEX | + | – | Severe |
| 55 | 3 or more doses | NHL | F | – | – | – | + | – | – | – | NA | NA | – | – | Mild |
| 59 | 2 doses | NHL | M | – | – | – | + | – | + | 12 | 11 | NIR/RIT | + | – | Moderate |
| 62 | Unknown | MDS | F | + | uPBSCT | -524 | – | – | – | – | 38 | NA | – | – | Mild |
| 62 | Unvaccinated | ALL | F | + | CBT | -671 | – | – | – | – | 26 | RDV | + | – | Mild |
| 68 | 2 doses | NHL | M | – | – | – | + | + | – | – | 39 | NIR/RIT | – | – | Mild |
| 69 | 1 dose | AML | M | + | CBT | -434 | – | – | – | – | 30 | RDV | + | – | Moderate |
| 73 | 3 or more doses | Others | M | – | – | – | – | – | – | – | 37 | RDV, DEX | + | – | Severe |
| 75 | 3 or more doses | AML | M | + | rPBSCT | -3643 | – | – | – | – | 1 | MOL | + | – | Mild |
| 79 | Unknown | MM | F | – | – | – | – | – | – | – | 16 | DEX | + | – | Severe |
| 82 | 3 or more doses | CLL | F | – | – | – | + | – | – | – | 17 | MOL, RDV, DEX | + | – | Severe |
| 85 | 3 or more doses | NHL | M | – | – | – | + | – | – | – | 4 | RDV | + | – | Mild |
Abbreviations: AML, acute myeloid leukemia; MDS, myelodysplastic syndromes; CML, chronic myeloid leukemia; ALL, acute lymphocytic leukemia; CLL, chronic lymphocytic leukemia; HL, Hodgkin lymphoma, NHL, non Hodgkin lymphoma; MM, multiple myeloma; HSCT, hematological stem cell transplantation; CBT, cord blood transplantation; rPBSCT, related peripheral blood stem cell transplantation; uPBSCT, unrelated peripheral blood stem cell transplantation; uBMT, unrelated bone marrow transplantation; CAR-T, chimeric antigen receptor-T; NIR/RIT, nirmatrelvir/ritonavir; RDV, remdesivir; MOL, molnupiravir; DEX, dexamethasone; BAR, baricitinib; Tix/Cil, tixagevimab-cilgavimab; NA, not available.
